# Mechanistic Insights on the Formation of a Carbodiimide Ion from Urea in La_2_O_2_NCN Synthesis Based on the “Proanion” Strategy

**DOI:** 10.1021/acs.inorgchem.4c02260

**Published:** 2024-08-05

**Authors:** Oomi Sumioka, Naoki Tarutani, Kiyofumi Katagiri, Kei Inumaru, Zi Lang Goo, Kunihisa Sugimoto, Yusuke Asai, Miwa Saito, Teruki Motohashi

**Affiliations:** † Graduate School of Advanced Science and Engineering, Hiroshima University, 1-4-1 Kagamiyama, Higashi-Hiroshima 739-8527, Japan; ‡ Department of Chemistry, Graduate School of Science and Engineering, Kindai University, 3-4-1 Kowakae, Higashi-Osaka 577-8502, Japan; § Department of Applied Chemistry, Faculty of Chemistry and Biochemistry, Kanagawa University, 3-27-1 Rokkakubashi, Kanagawa-ku, Yokohama 221-8686, Japan

## Abstract

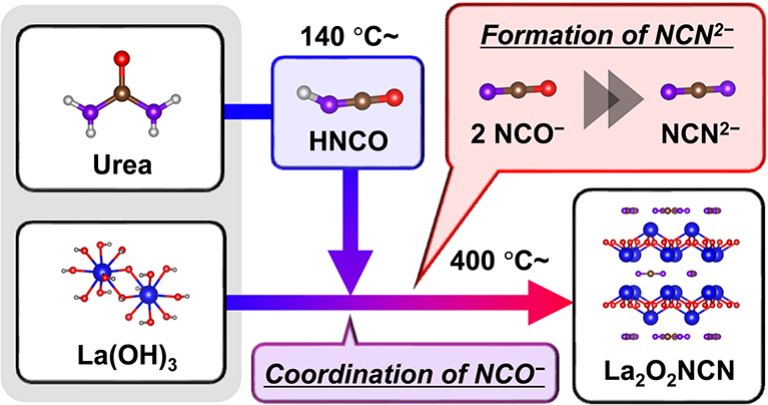

This study affords mechanistic insights into the formation mechanism of carbodiimide ions (NCN^2–^) from urea during the synthesis of La_2_O_2_NCN by employing the “proanion” strategy without using NH_3_ gas. It is a safer, cost-effective, and environmentally friendly approach. Urea, acting as a proanion, decomposes upon heating, facilitating conversion to NCN^2–^. This work meticulously examines the phase transitions and identifies intermediate species formed during the reaction using *in situ* X-ray diffraction, Fourier transform infrared spectroscopy, and thermogravimetric–differential thermal analysis–mass spectrometry. The findings present a detailed mechanism in which urea initially decomposes at 140 °C, releasing HNCO, which reacts with La(OH)_3_ to immobilize NCO^–^ species on the surface of La(OH)_3_. As the temperature reaches approximately 400 °C, these NCO^–^ anions transform into NCN^2–^ anions by releasing CO_2_ gas, resulting in the formation of an amorphous phase rich in NCN^2–^. Following further heating to 600 °C, La_2_O_2_NCN crystallizes, enhancing its crystallinity as the temperature increases. These findings elucidate the formation mechanism of La_2_O_2_NCN, introduce the “proanion method” for the alternative synthesis of metal (oxy)carbodiimides, and expand their potential for applications as functional materials.

## Abstract 2

This study elucidates the formation mechanism of carbodiimide ions from urea during the synthesis of La_2_O_2_NCN by employing the “proanion” strategy. *In situ* measurements reveal that the process involves urea decomposition to NCO^−^, interaction with La(OH)_3_, and subsequent transformation from NCO^−^ to NCN^2−^ at elevated temperatures, culminating in La_2_O_2_NCN crystallization. This approach broadens the potential of metal (oxy)carbodiimides as functional materials.

## Introduction

Ceramic materials, epitomized by metal oxides, are pivotal inorganic solid materials that permeate various aspects of our daily life, from the mundane, such as tableware, to more sophisticated applications, such as components in electronic devices. The exploration of ceramic materials has evolved from focusing solely on single-anion entities, such as metal oxides, to delving into the realm of mixed-anion compounds, including metal oxynitrides, which contain multiple types of anions in a single material.^1,2^ However, the advancements have largely remained confined to materials composed of monatomic ions, even with mixed-anion compounds. Recently, the investigation of materials that harbor both monatomic and molecular ions has become a burgeoning field of interest within the research of ionic crystal-type inorganic solids.^3−5^ This new class of materials is garnering significant attention due to its unique characteristics, such as anisotropy, alignment, and dynamic behaviors, including vibration, rotation, and intramolecular reactions, conferred by the molecular ions. These attributes, absent in monatomic ions, facilitate the manifestation of novel functionalities.

Quintessential examples of this innovative direction include studies on metal carbodiimides that incorporate the NCN^2–^ anion.^6−14^ NCN^2–^ exhibits tautomerism between its symmetric carbodiimide ([N=C=N]^2–^) and asymmetric cyanamide ([N—C≡N]^2–^) forms, offering the potential for unique functions that cannot be achieved with monatomic ions. For example, Ag_2_NCN has been proposed as a potential visible-light-responsive photocatalytic material based on a model in which the tautomerism of NCN^2–^ effectively separates photoexcited electrons and holes.^8^ Moreover, metal (oxy)carbodiimides are recognized for their potential to be hosts for luminescent materials. Masubuchi et al. demonstrated that BaNCN:Eu^2+^, a red luminescent material, exhibits a pressure-induced fluorescence wavelength shift that surpasses that of ruby, the traditional benchmark for pressure sensors, by approximately 50-fold.^13^ Additionally, in research on rare-earth oxycarbodiimides, *RE*_2_O_2_NCN (*RE* = rare earths) has made advances as hosts for luminescent materials with potential applications in fluorescence and upconversion luminescent materials.^15−23^*RE*_2_O_2_NCN has a tetragonal (*RE* = La and Ce) or a trigonal (*RE* = except for La) structure, which is analogous to *RE*_2_O_2_*Ch* (*Ch* = S, Se, and Te), comprised of alternating [*RE*_2_O_2_]^2+^ layers and rodlike NCN^2–^ anions arranged in parallel or perpendicularly to the *c* axis.^15,16^ NCN^2–^ is a nitrogen-based pseudochalcogenide ion that lies between the atoms of O^2–^ and S^2–^ from the hard/soft acid/base perspective. Because the structure and divalent anion of *RE*_2_O_2_NCN is related to those of *RE*_2_O_2_Ch, these materials exhibit light-emitting properties with doping activator ions such as Eu^3+^, Tb^3+^, and Er^3+^/Yb^3+^.^18−23^ Our previous study reported that La_2_O_2_NCN:Eu^3+^ could luminescence with an excitation light of longer wavelength by broadening its excitation wavelength region in comparison with La_2_O_3_:Eu^3+^.^23^

Generally, such metal (oxy)carbodiimides are synthesized using the solution process,^8,24^ solid-state metathesis method,^7,14,25^ or calcination of precursors under a stream of NH_3_ gas.^12,13,15−18,21,22^ Although solid-state metathesis is straightforward, it occasionally necessitates vacuum sealing in ampules and is unsuitable for systems with easily reducible metal elements. The reliance on a highly toxic and expensive reagent is a serious problem of the ammonia gas method, which is compounded by the need for a prolonged reaction time and a high temperature. These obstacles hinder further developments of research on metal (oxy)carbodiimides, including *RE*_2_O_2_NCN, as functional materials.

In this context, we recently reported the preparation of La_2_O_2_NCN via the heat treatment of a mixture of La(OH)_3_ and urea, eliminating the need for NH_3_ gas.^23^ Urea serves as a precursor that decomposes to the NCN^2–^ anion during heating. Compounds that convert into the desired form during the reaction are often represented by the prefix of “pro-”, such as provitamin^26^ and prochirality.^27^ Following this, we define compounds that transform to the desired molecular anion via the reaction as “proanion”. The “proanion method” is a significant strategy to synthesize *RE*_2_O_2_NCN due to its simple process and low cost and because it is a safe alternative to the conventional method of using NH_3_ gas. However, the conversion process from urea to the NCN^2–^ anion remains unresolved, and it is crucial to elucidate the reaction mechanism of the proanion method to establish an alternative synthesis process for *RE*_2_O_2_NCN.

This study explored the formation mechanism of La_2_O_2_NCN by the solid-state method using urea as a proanion. First, *in situ* X-ray diffraction (XRD) measurements were performed to observe the phase transition of the precursor during heat treatment. Subsequently, *in situ* Fourier transform infrared (FT-IR) spectroscopy and thermogravimetric–differential thermal analysis–mass spectrometry (TG–DTA–MS) measurements were performed to identify the intermediate species formed during the process. Finally, we elucidated the reaction mechanism of the proanion method using urea.

## Experimental Section

### Materials

Lanthanum nitrate hexahydrate [La(NO_3_)_3_·6H_2_O, 99.9%], urea (CO(NH_2_)_2_, 99%), and an aqueous solution of sodium hydroxide (NaOH aq., 5 mol dm^–3^) were purchased from Nacalai Tesque, Inc. (Kyoto, Japan). All of the reagents were used as received without further purification. A Milli-Q purification system (Merck Millipore, Billerica, MA) was used to purify the deionized (DI) water used in all experiments.

### Preparation of La_2_O_2_NCN Using Urea as a Proanion

La_2_O_2_NCN was prepared by following a previously reported method.^23^ La(OH)_3_, used as a precursor of La_2_O_2_NCN, was prepared via a hydrothermal method. La(NO_3_)_3_·6H_2_O (4.83 mmol) was dissolved in DI water (60 mL), and NaOH aq. (7.7 mL) was added to the solution under vigorous stirring. Following stirring for 30 min, the suspension was transferred to a Teflon vessel. The vessel was sealed in a stainless-steel autoclave and heated at 200 °C for 24 h. Upon cooling, the product was washed with DI water three times and dried overnight in an oven at 70 °C. The La(OH)_3_ obtained was mixed with urea, used as a proanion for the carbodiimide ion in La_2_O_2_NCN. The molar ratio of urea to La(OH)_3_ in the mixture was fixed to 2.5. The mixture was heat-treated using an alumina crucible boat under a N_2_ stream (500 mL min^–1^) in a horizontal tube furnace. The furnace was heated at a rate of 15 °C min^–1^ and maintained at 800 °C for 2 h.

### Characterizations

XRD measurements were performed by using a D2 Phaser X-ray diffractometer (Bruker AXS, Karlsruhe, Germany) with Cu Kα radiation to identify the crystalline structures of the obtained samples. Various *in situ* measurements were performed to directly evaluate the process by which the proanion (i.e., urea) converted to an anion (i.e., NCN^2–^) during the formation of La_2_O_2_NCN. The measurements were performed using a mixture of the precursor [La(OH)_3_] and proanion with the same molar ratio as that shown in the preparation conditions given above. *In situ* XRD measurements were performed at the BL02B2 beamline in the SPring-8 synchrotron facility (Hyogo, Japan) using λ = 0.5 Å radiation to monitor the phase transitions of crystalline species in a mixture of the precursor and proanion during the heat-treatment process. A flat-panel detector was selected to observe continuous dynamic changes quickly. The mixture (approximately 0.8 mg) was placed in a sapphire cell and covered with a sapphire lid with a hole on it. Prior to measurement, the sapphire cell was purged with N_2_ gas (100 mL min^–1^) for 30 min. During the *in situ* measurement, the cell was heated at a rate of 15 °C min^–1^ up to a plateau temperature of 650 °C under a N_2_ stream (10 mL min^–1^). To identify molecular species generated as intermediates during heat treatment, *in situ* FT-IR spectra were recorded over the range of 1000–4000 cm^–1^ using an FT/IR-4700 spectrometer (JASCO, Tokyo, Japan) with DR-650Ai (JASCO, Tokyo, Japan) for heating diffuse-reflectance spectroscopy (DRS) analysis. During *in situ* measurement, the cell was heated at a rate of 10 °C min^–1^ and maintained at 800 °C for 2 h under a N_2_ stream (100 mL min^–1^). Collected *in situ* IR data were analyzed using spectroscopic manager software (JASCO, Tokyo, Japan) without fixing the peak position, half-width, height, and fitting function. To elucidate the thermal behavior of the samples and the chemical nature of released gases during the heat-treatment process, TG–DTA–MS was performed using an STA-449 (NETZSCH, Selb, Germany) combined with JMS-Q1500GC (JEOL, Tokyo, Japan). Data acquisition was performed only during the heating process. The sample was heated linearly at a heating rate of 10 °C min^–1^, and He gas (flow rate: 20 mL min^–1^) was used to provide the inert atmosphere for measurements. The detection range was set as *m*/*z* 10–500. Scanning electron microscopy (SEM; Hitachi S-4800) was performed to observe the morphology of the obtained samples. The UV–vis diffuse-reflectance spectrum (DRS) of La_2_O_2_NCN was recorded using a V-750 spectrophotometer (JASCO, Tokyo, Japan). The oxygen, carbon, and nitrogen contents were determined by wavelength-dispersive X-ray fluorescence (XRF) spectroscopy (ZSX Primus IV, Rigaku, Tokyo, Japan).

## Results and Discussion

An XRD pattern and an FT-IR spectrum of the sample obtained from a mixture of La(OH)_3_ and urea after heat treatment at 800 °C for 2 h are shown in Figure S1. The diffraction peaks are attributable to La_2_O_2_NCN (tetragonal phase, space group *I*4/*mmm*), wherein linear carbodiimide anions [N=C=N]^2–^ positioned in parallel with [La_2_O_2_]^2+^ cation layers are shown in the XRD pattern (Figure S1a). Diffraction peaks attributed to La_2_O_3_, a byproduct, were also detected, albeit at a slight intensity. Lattice constants of the obtained La_2_O_2_NCN were calculated as *a* = 4.0986(1) Å and *c* = 12.340(4) Å. These values are well consistent with those previously reported by Hashimoto and co-workers.^15^ A FT-IR spectrum of the sample displays two absorption bands: asymmetric stretching (ν_as_ 1960 cm^–1^) and deformation (δ 681 cm^–1^) vibrations of NCN^2–^ (Figure S1b).^28,29^ Judging from the absence of a symmetric stretching vibration (approximately 1250 cm^–1^), which is IR-forbidden for carbodiimide [N=C=N]^2–^, NCN^2–^ anions exist in the carbodiimide states in the obtained La_2_O_2_NCN. These band assignments are consistent with previous works on La_2_O_2_NCN and other metal carbodiimides, such as *M*_2_O_2_NCN (*M* = Bi, Sc, and Sn).^24,25,30,31^ UV–vis DRS revealed that the absorption edge of the obtained La_2_O_2_NCN exists at around 260 nm (Figure S1c). This value of the absorption edge position is comparable to that reported in a preceding paper by another group.^17^ SEM observation represents that the obtained La_2_O_2_NCN exhibits an undefined morphology with sizes generally below 500 nm (Figure S1d). According to the wavelength-dispersive XRF measurements, the molar ratio of O, C, and N in the obtained La_2_O_2_NCN was 2.1:1:2.2, which was almost consistent with the theoretical value (O:C:N = 2:1:2). These results reveal that La_2_O_2_NCN could be synthesized in almost a single phase under the conditions, as outlined in our previous report.^24^ It is evident that an optimal mixing ratio of the precursor, La(OH)_3_, proanion, and urea and optimal heat-treatment conditions (temperature and time) are needed to synthesize La_2_O_2_NCN in a single phase. However, the process by which urea is converted to NCN^2–^ has not been clarified.

**Figure 1:**
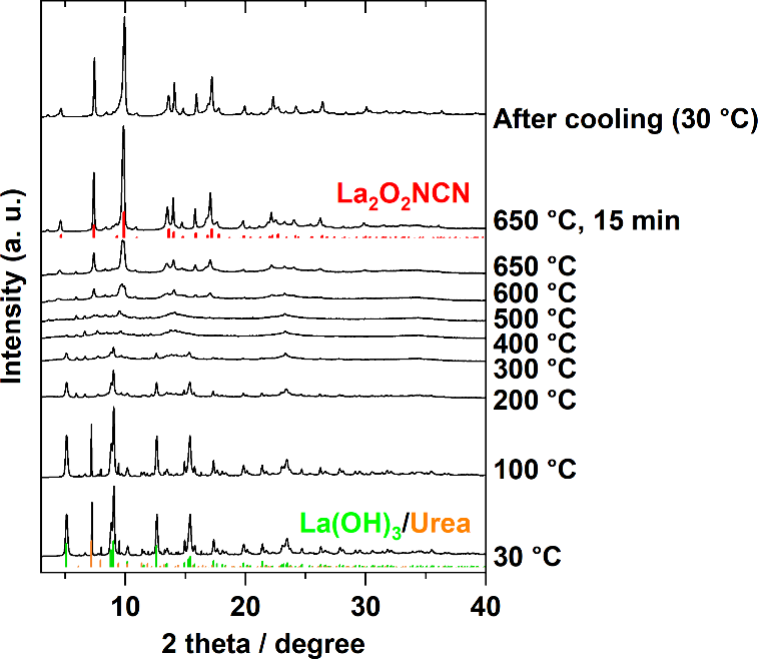
*In situ* XRD patterns of a mixture of La(OH)_3_ and urea during the heating process from 30 to 650 °C (λ = 0.5 Å).

To directly evaluate the reaction process, it is necessary to directly analyze the state in which the sample is in when it reaches each temperature using *in situ* measurement techniques. Therefore, *in situ* XRD, *in situ* FT-IR, and TG–MS measurements were performed under the reaction conditions described above. Figure 1 illustrates the *in situ* XRD patterns upon heating from room temperature to 650 °C. In the XRD pattern of the sample at the start of the measurement (30 °C) and when the temperature reached 100 °C, the detected diffraction peaks can be assigned to La(OH)_3_ and urea. The peaks attributed to urea disappeared when the temperature reached 140 °C due to thermal decomposition (Figure S2).^32^ When the sample temperature exceeds 200 °C, the peak intensity of La(OH)_3_ decreases and almost disappears at 400 °C, indicating that most of the sample turned into an amorphous state. Once the heating temperature of the sample exceeded 600 °C, diffraction peaks attributed to La_2_O_2_NCN appeared and became more evident at 650 °C. When the sample was held at this temperature for 15 min, the diffraction peaks increased in intensity and became sharper. Thereafter, no significant change in the peak intensity was observed in the XRD pattern measured after heating was stopped and the sample temperature decreased to 30 °C. Therefore, this indicates that La_2_O_2_NCN formed and crystallized during the heating process rather than during cooling. The heating temperature of 600 °C is the critical temperature to crystallize the La_2_O_2_NCN phase, particularly considering the longer duration *in situ* and *ex situ* measurements (Figure S3a). These results reveal that La_2_O_2_NCN formed through an intermediate state of the amorphous species during heating.

**Figure 2:**
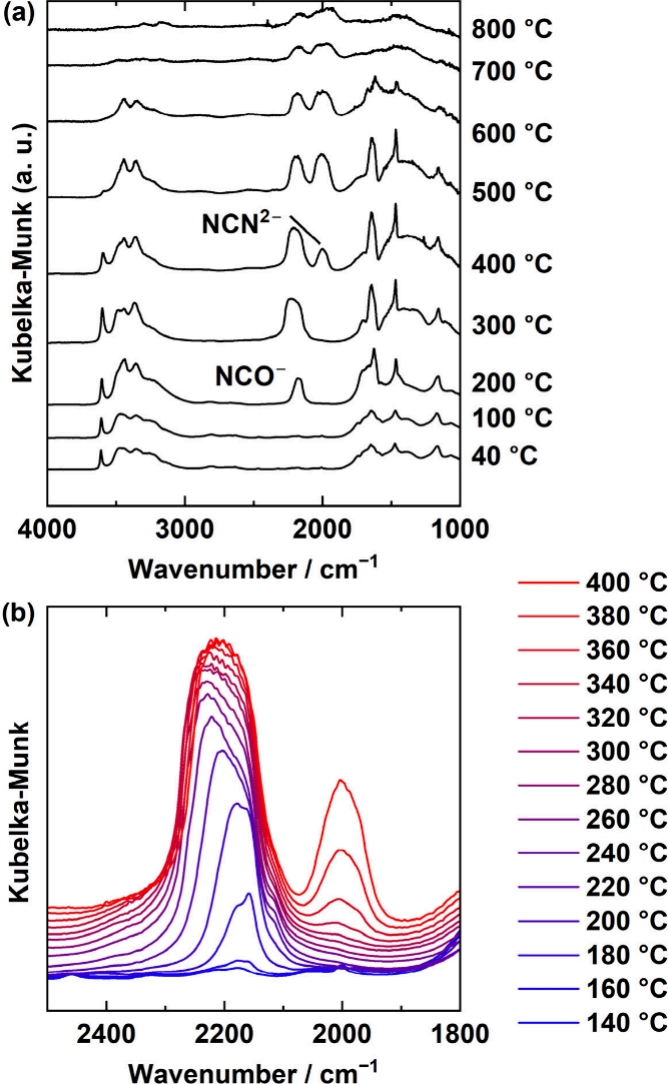
*In situ* FT-IR DRS of a mixture of La(OH)_3_ and urea when heated from (a) 40 to 800 °C, measured every for 100 °C, and (b) 140 to 400 °C, measured every 20 °C.

We performed *in situ* FT-IR measurements to investigate the reaction processes during the amorphous phases including the chemical species generated upon thermal decomposition of urea. Additionally, TG–DTA–MS measurements were performed for thermal analysis during the reaction process and to identify the chemical species in the gas released during the reaction. Figure 2 illustrates *in situ* FT-IR spectra during the heating process from 40 to 800 °C. The absorption bands in the spectra of the sample until the heating temperature reaches 140 °C match those detected in the spectra of La(OH)_3_ and urea alone (Figure S4). Therefore, it is clear that the sample is a mixture of La(OH)_3_ and urea up to this temperature. As the heating temperature increases, characteristic spectral changes are observed in the wavenumber region from 2300 to 1900 cm^–1^. The detailed results of spectral measurements performed at 20 °C intervals in the range of 140–400 °C in this wavenumber region are depicted in Figure 2b. The absorption band at approximately 2150 cm^–1^ appears above 140 °C, indicating that the band is derived from the species generated by the decomposition of urea. A previous study reported that urea decomposed to gaseous NH_3_ and isocyanic acid (HNCO) in an inert atmosphere at 140 °C, as described in the following reaction equation:^32^
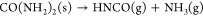
1

**Figure 3:**
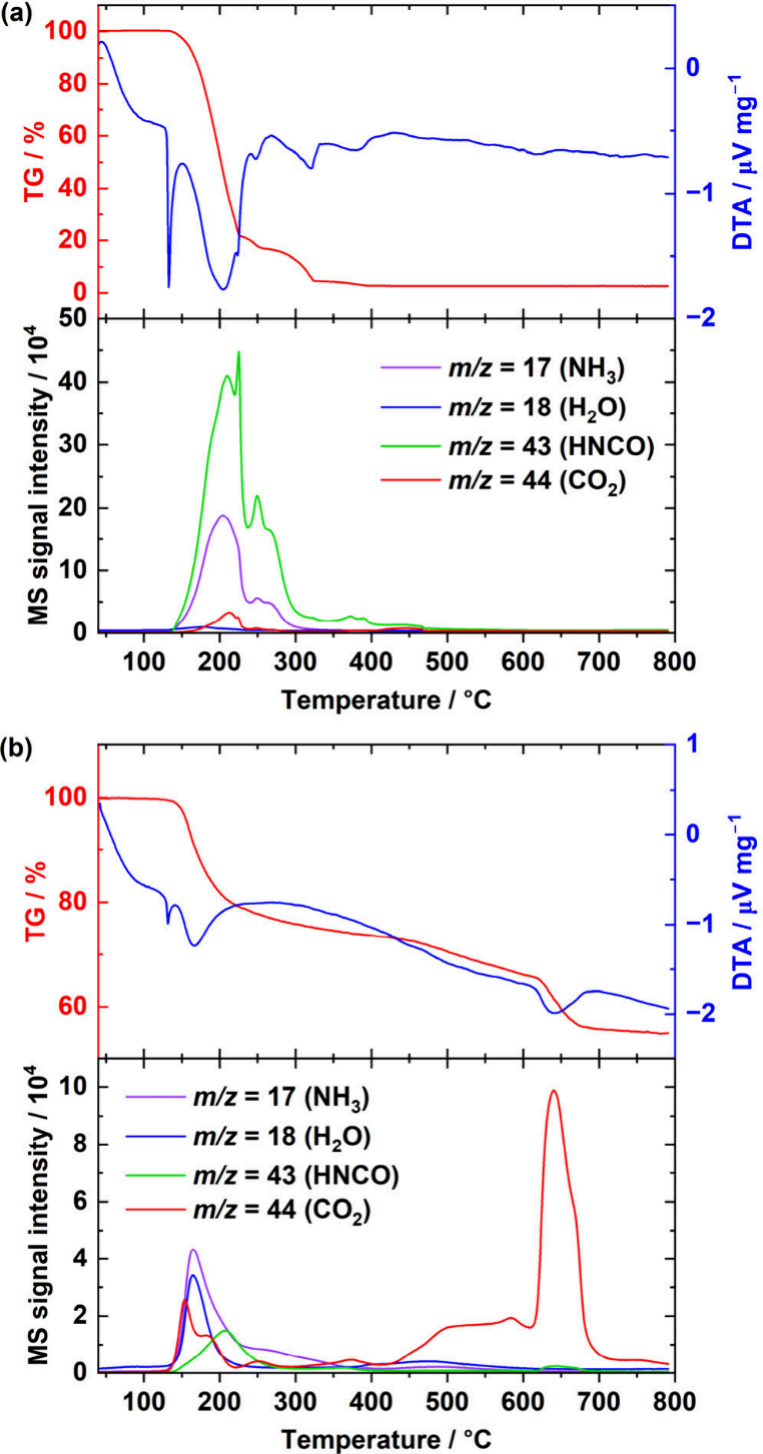
TG–DTA (upper panel) and MS (lower panel) curves of (a) urea and (b) a mixture of La(OH)_3_ and urea from 40 to 800 °C.

The wavenumber region of the absorption band at approximately 2150 cm^–1^ is inherent to an asymmetric stretching vibration mode (ν_as_) of the NCO^–^ group. In general, the thermal decomposition of urea alone results in the formation of more complex species, such as biuret and cyanuric acid, through reactions (2) and (3),^32^ but the absorption bands assigned to these species are not found in *in situ* FT-IR spectra (Figure S4).


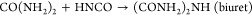
2
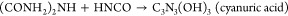
3Because the boiling point of HNCO is 23.5 °C,^33^ it should be in a gas state when present alone. *In situ* measurements were performed under a N_2_ gas flow; therefore, if HNCO existed in a gaseous state, the NCO^–^ band would not be detected in the IR spectrum. This indicates that HNCO generated by the thermal decomposition of urea was trapped on the surface of La(OH)_3_. The thermal decomposition behaviors of urea alone and in the presence of La(OH)_3_ were compared using TG–DTA–MS measurements, as presented in Figure 3. In the case of urea alone, a sharp endothermic peak without weight loss is found at 130 °C, derived from the melting of urea (Figure 3a). Weight loss started at 140 °C, with an endothermic signal representing the thermal decomposition of urea. Strong signal intensities of NH_3_ (*m*/*z* 17) and HNCO (*m*/*z* 43) at approximately 200 °C indicate that urea decomposed through reaction (1). In the case of a mixture of La(OH)_3_ and urea, the TG–DTA results are comparable to those of urea alone in this temperature range; however, a clear difference is observed in the MS measurement (Figure 3b). Compared with the case with urea alone, the relative signal intensity of HNCO (*m*/*z* 43) at approximately 200 °C was clearly suppressed. This suggests that the NCO^–^ species detected in the FT-IR spectrum is generated by the thermal decomposition of urea and is not released outside the reaction system as a gas species; however, it is immobilized by interacting with the La(OH)_3_ surface.

Subsequently, once the heating temperature reaches 400 °C, at which an absorption band appears at around 2000 cm^–1^, the chemical species in the *in situ* FT-IR spectra (Figure 2) are focused. This absorption band can be assigned to the asymmetric stretching of NCN^2–^. In the *in situ* XRD measurements (Figure 1), almost no peaks are detected in this temperature range, so it appears that NCN^2–^ anions are generated in an amorphous state. In the TG–DTA–MS measurements, almost no MS signal was detected at higher than 400 °C for urea alone. In contrast, signals of H_2_O (*m*/*z* = 18) and CO_2_ (*m*/*z* 44) were observed above 400 °C for a mixture of La(OH)_3_ and urea. Focusing on changes in the intensity of the MS signal of CO_2_, it can be observed that the signal begins to appear at approximately 400 °C, becomes stronger after 600 °C, and reaches its maximum at 650 °C. Furthermore, it was found that the release of CO_2_ from the sample almost stops at approximately 700 °C. In *in situ* FT-IR measurements, the temperature range of 400 °C, where the MS signal begins to appear, coincided with the temperature range in which the NCO^–^ absorption band decreases and the NCN^2–^ absorption band begins to appear. In the temperature range of 600–700 °C, where the MS signal of CO_2_ is strongly observed, a weight loss of more than 10% was found in the TG results of a mixture of La(OH)_3_ and urea. Additionally, based on the results of *in situ* XRD at 600–700 °C, La_2_O_2_NCN generated and crystallized in this temperature range. Considering these results comprehensively, it can be concluded that, in the reaction system, two NCO^–^ ions adsorbed on the La precursor undergo a thermal reaction, and one NCN^2–^ ion is produced with the release of CO_2_ as a byproduct as follows:
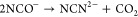
4

**Figure 4:**
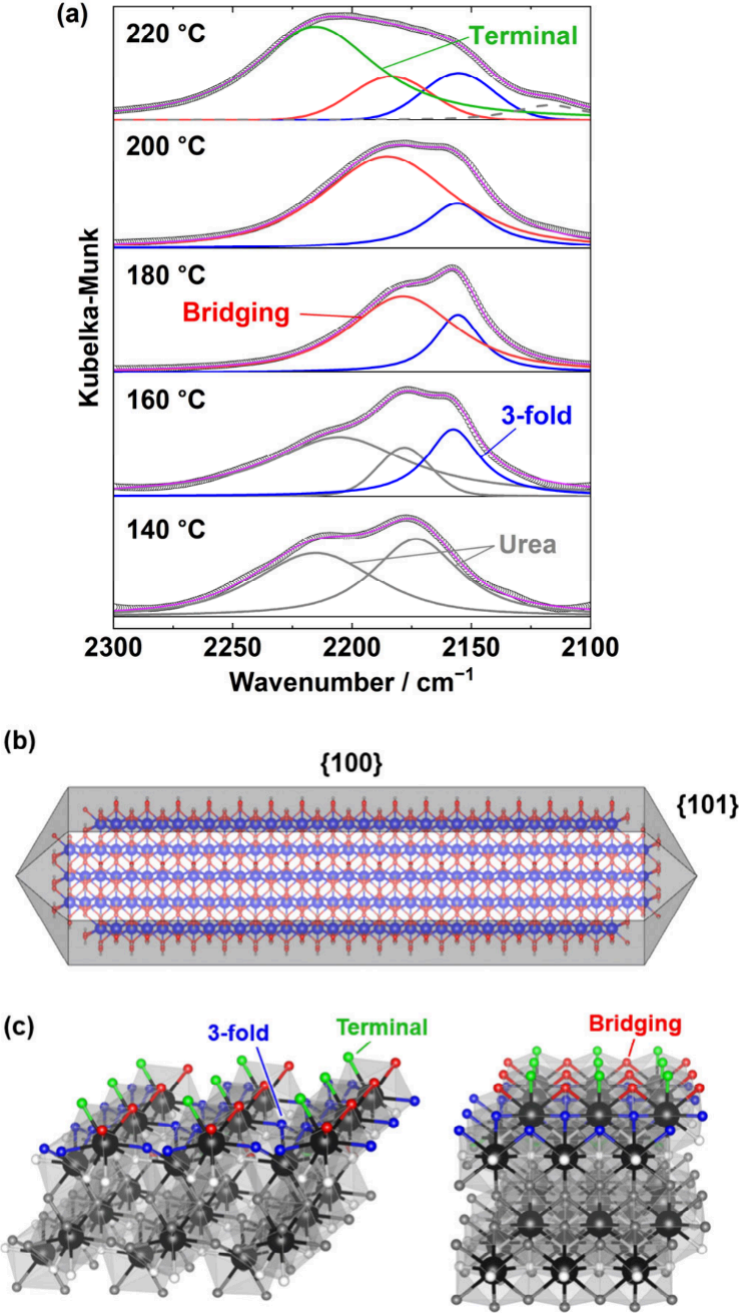
(a) *In situ* FT-IR spectra from 140 to 220 °C: raw data (circle) with fitted curves (solid line). Green, red, blue, and gray lines represent “terminal”, “bridging”, and “3-fold” NCO^–^ species and urea, respectively. The gray dashed line at 220 °C represents unidentified species. (b) 3D model of the rodlike La(OH)_3_ particle. (c) Exposed {100} plane of La(OH)_3_. Green, red, and blue atoms represent “terminal”, “bridging”, and “3-fold” O atoms in OH^–^ anions, respectively.

The chemical bonding nature of intermediates containing NCO^–^ was further investigated. Figure 4a illustrates the *in situ* FT-IR spectra fitting data that cover a wavenumber region of 2300–2100 cm^–1^, observed from 140 to 220 °C. Between 140 and 160 °C, an absorption band appears at approximately 2160 cm^–1^, which could not be assigned to urea. Upon increasing the heating temperature to 180 and 220 °C, additional bands emerged at 2180 and 2220 cm^–1^, respectively. This suggests that the NCO species in this reaction system exists in three distinct chemical states. The observed wavenumbers for these states are all lower than that of gaseous HNCO (2254 cm^–1^),^34^ implying a strong interaction between the NCO species and La(OH)_3_. The detection of the H_2_O (*m*/*z* 18) signal at 130 °C (Figure 3b), which is lower than the dehydration temperature of La(OH)_3_ (310 °C),^35^ implies that the surface OH ligand of La(OH)_3_ and HNCO can be expressed as follows:
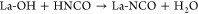
5Here, NCO^–^ anions tend to bond with metal cations through N atoms, forming La–N bonds, due to their significant negative partial charge.^36^ The rod-shaped La(OH)_3_ precursor, as shown in Figure S5b, exposes its {100} planes (Figure 4b), as supported by the previous study.^37^ On this facet, the exposed OH ligands display various states classified as coordinated by one, two, and three La^3+^ cations (defined as “terminal”, “bridging”, and “3-fold”, respectively; Figure 4c). These states appear to be the origin of the three distinct adsorption bands observed in the IR spectra. The wavenumber position of ν_as_(NCO^–^) of ML_2_(NCO)_2_ (“bridging” NCO^–^) was reported to be higher than that of ML_2_(NCO)_4_ (“terminal” NCO^–^).^38^ Another study simulatively showed that the wavenumber position of the ν_as_(NCO^–^) band of adsorbed NCO on the Pt(001) facet decreases as its coordination number increases.^39^ Drawing from these insights, absorption bands located at approximately 2220, 2180, and 2160 cm^–1^ are identified as corresponding to “terminal”-, “bridging”-, and “3-fold”-coordinated NCO^–^ anions, respectively. In brief, the coordination of NCO^–^ species proceeds as follows: NCO^–^ anions start to coordinate to the “3-fold” sites at 160 °C, to “bridging” sites at 180–200 °C, and to “terminal” sites at temperatures of 220 °C or higher. The intensity of the absorption bands for coordinated NCO^–^ species exceeds 220 °C and begins to decrease from 360 °C, as shown in Figure 2b, and the NH_3_ signal is not observed in the MS curve of the mixture of La(OH)_3_ and urea in Figure 3b, i.e., supplying HNCO through reaction (1). These results are consistent with the aforementioned analyses and suggest a significant role of NCO^–^ anions in the formation of La_2_O_2_NCN.

**Scheme 1:**
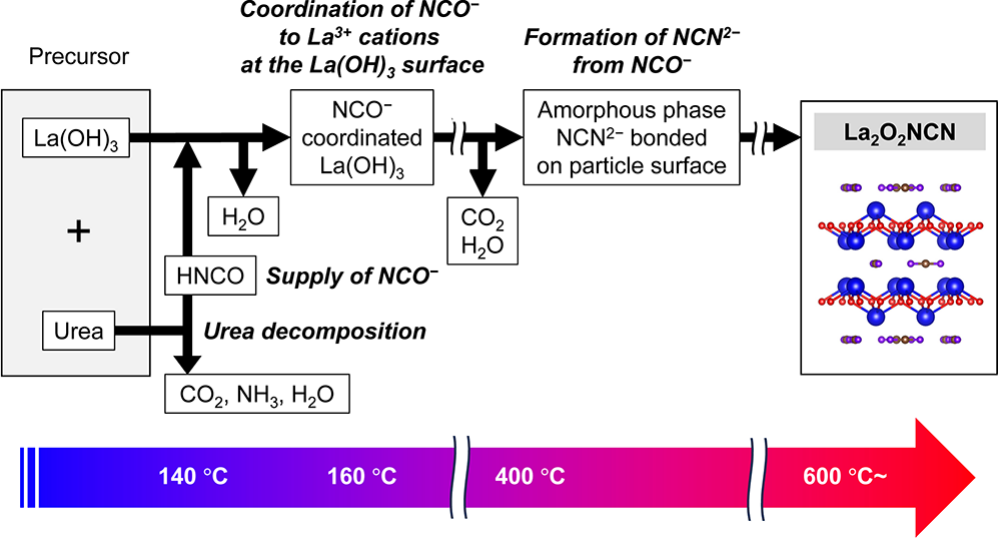
Formation Mechanism of La_2_O_2_NCN Using Urea as a Proanion

On the basis of our analyses using *in situ* XRD, *in situ* FT-IR, and TG–DTA–MS, we propose a formation mechanism of La_2_O_2_NCN using urea as a proanion (Scheme 1). Initially, urea decomposes at 140 °C, releasing HNCO. The released HNCO then reacts with OH ligands in La(OH)_3_ to form three types of NCO^–^ species. Once the temperature reaches approximately 400 °C, these NCO^–^ anions start to transform into NCN^2–^ anions. The process results in the formation of the amorphous phase containing NCN^2–^, along with the release of CO_2_. When the temperature further increases to 600 °C, La_2_O_2_NCN begins to crystallize, and its crystallinity continues to improve with the temperature.

## Conclusions

In this study, we elucidated the intricacies of the formation mechanism of La_2_O_2_NCN through a solid-state reaction using urea as a proanion. The unique characteristics of a urea proanion were leveraged to employ various *in situ* analytical techniques to uncover the conversion from urea to carbodiimide anions during the formation of La_2_O_2_NCN. HNCO gases generated during the thermal decomposition of urea engaged in a chemical reaction with the hydroxyl OH ligands on the surface of La(OH)_3_ to form La^3+^–NCO^–^ bonds, marking the first critical step in the synthesis pathway. A transformation can be imitated by heating to approximately 400 °C, where the previously formed NCO ligands convert to NCN^2–^ anions, which enable the formation of an intermediate amorphous phase containing NCN^2–^. As the temperature exceeds 600 °C, this intermediate undergoes crystallization, culminating in the formation of La_2_O_2_NCN. The significance of the findings lies in elucidating the formation mechanism of La_2_O_2_NCN and introducing a method that is more efficient, safer, and environmentally sustainable than conventional ammonolysis. The traditional ammonolysis method relies on the high-temperature reaction of precursor oxides with hazardous NH_3_ gas, typically at temperatures close to 950 °C in a graphite boat. In addition to the significant safety and environmental concerns, it limits scalability and efficiency due to high energy demand. In contrast, our proanion approach operates at substantially lower temperatures without ammonia gas, thereby reducing both the environmental impact and synthesis costs. We believe that the proanion method will enable us to access various other metal (oxy)carbodiimides as well as *RE*_2_O_2_NCN and contribute to accelerating the development of the chemistry of metal (oxy)carbodiimides as functional materials.

## Supporting Information Available

XRD pattern, FT-IR spectrum, UV–vis DRS, and SEM image of La_2_O_2_NCN prepared by using urea as a proanion, *in situ* XRD patterns of the precursor during the heating process from 100 to 200 °C, *ex situ* XRD patterns and *ex situ* FT-IR spectra of the precursor, FT-IR spectra of La(OH)_3_, urea, biuret, and cyanuric acid, and XRD pattern and SEM images of La(OH)_3_ prepared using the hydrothermal method (PDF)
