# Supplementary material for: Mechanistic Insights on the Formation of a Carbodiimide Ion from Urea in La2O2NCN Synthesis Based on the “Proanion” Strategy
Source: Inorg Chem. 2024 Aug 5;63(34):15539–45. doi: 10.1021/acs.inorgchem.4c02260 (PMC11351172; doi:10.1021/acs.inorgchem.4c02260)
Supplement: Supplementary file 1 — ic4c02260_si_001.pdf [file ic4c02260_si_001.pdf]

– Supporting Information –

Mechanistic Insights on the Formation of Carbodiimide Ion from Urea in La_2O_2NCN Synthesis Based on the "Proanion" Strategy

Oomi Sumioka,[†] *Naoki Tarutani,*[†] *Kiyofumi Katagiri,*[†,*] *Kei Inumaru,*[†] *Zi Lang Goo,*[‡] *Kunihisa Sugimoto,*[‡] *Yusuke Asai,*[§] *Miwa Saito,*[§] *and Teruki Motohashi*[§]

† Graduate School of Advanced Science and Engineering, Hiroshima University, 1-4-1 Kagamiyama, Higashi-Hiroshima 739-8527, Japan.

‡ Department of Chemistry, Graduate School of Science and Engineering, Kindai University, 3-4-1 Kowakae, Higashi-Osaka 577-8502, Japan.

§ Department of Applied Chemistry, Faculty of Chemistry and Biochemistry, Kanagawa University, 3-27-1 Rokkakubashi, Kanagawa-ku, Yokohama 221-8686 , Japan.

* To whom correspondence should be addressed. E-mail: kktgr@hiroshima-u.ac.jp

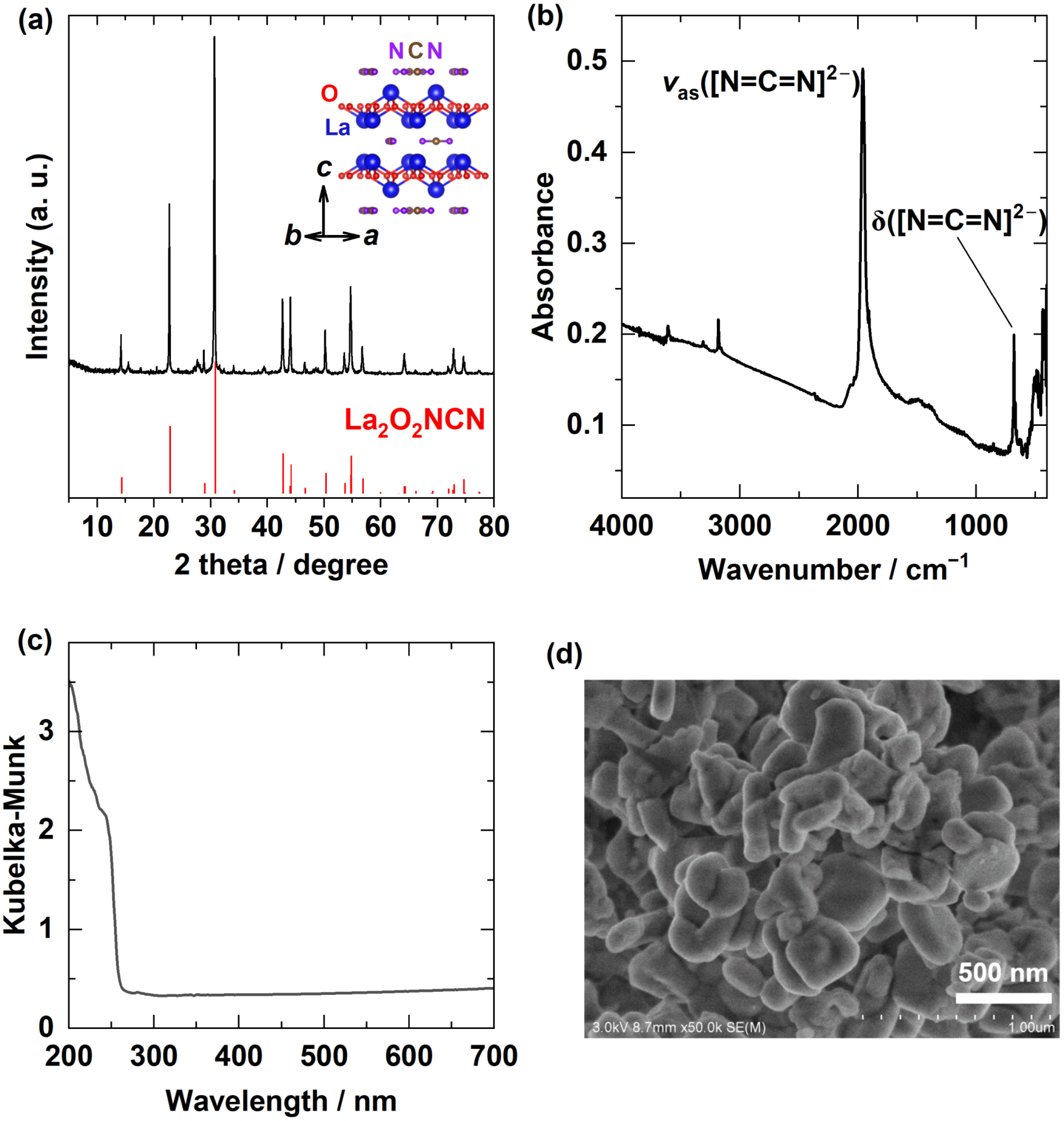

Figure S1. (a) XRD pattern (λ = Cu Kα), (b) FT-IR spectrum, (c) UV-vis-DRS, and (d) SEM image of La_2O_2NCN prepared by heat-treatment of the mixture of $La(OH)_3$ and urea at 800 °C for 2 h.

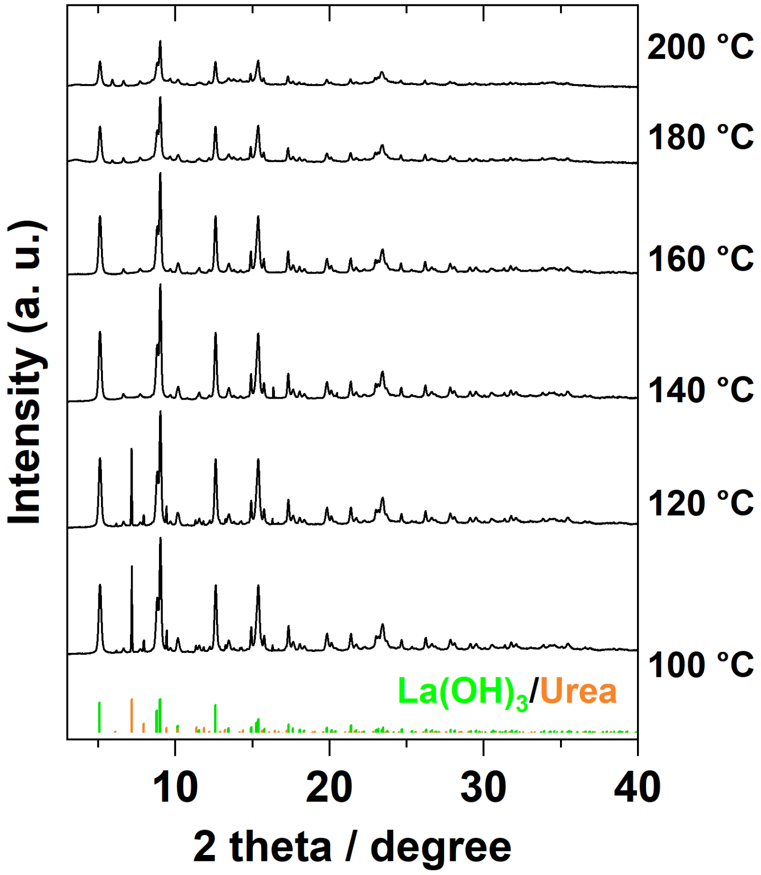

Figure S2. *In*-situ XRD pattern changes of the mixture of $La(OH)_3$ and urea during heating process from 100 °C to 200 °C (λ = 0.5 Å).

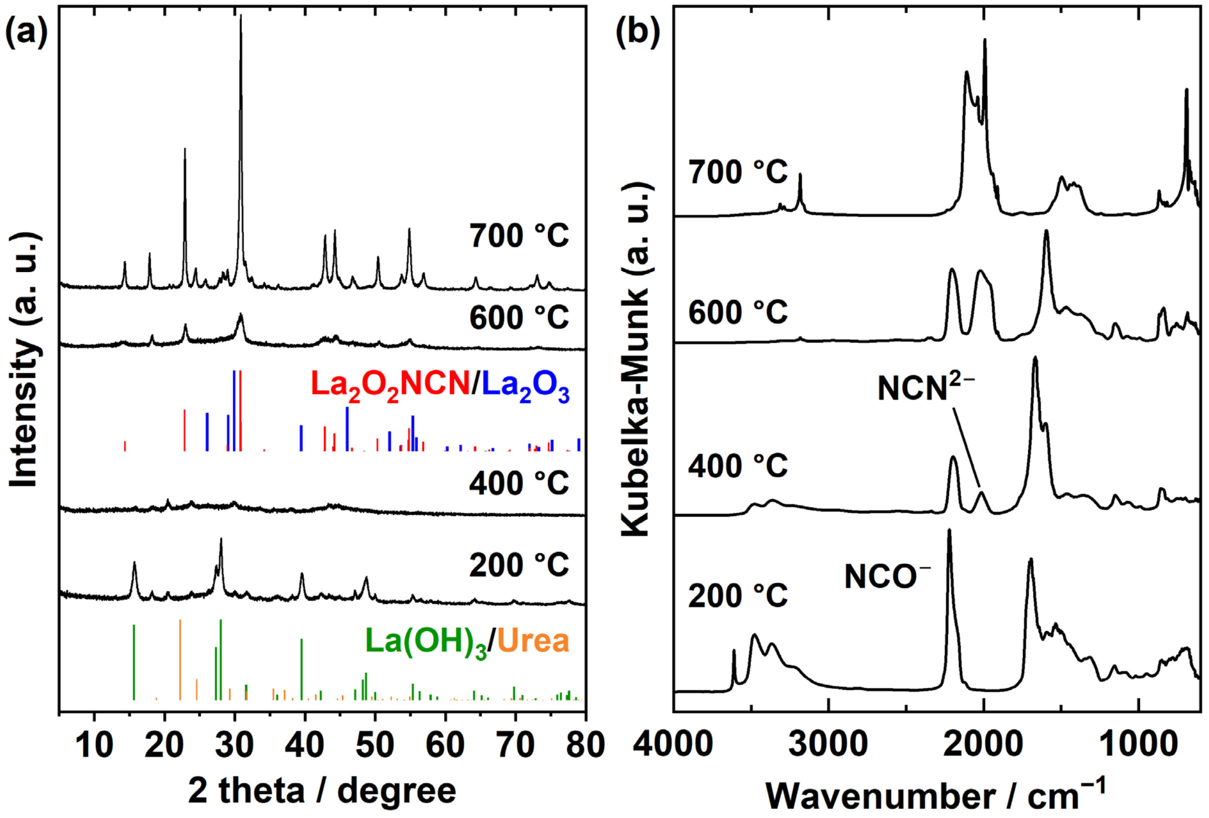

Figure S3. (a) *Ex-situ* XRD patterns and (b) *ex-situ* FT-IR spectra of the mixture of $La(OH)_3$ and urea thermally treated at various final temperatures (200 °C, 400 °C, 600 °C, and 700 °C) without duration processes.

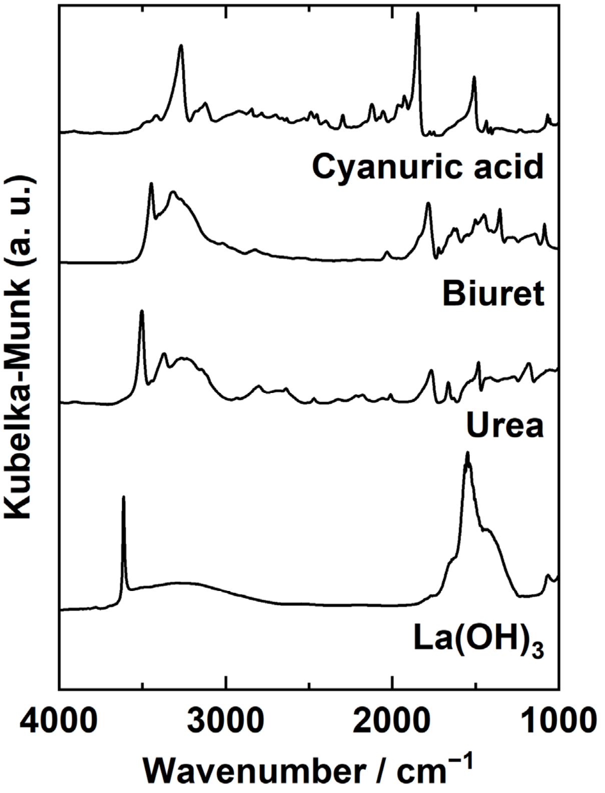

Figure S4. FT-IR spectra of $La(OH)_3$, urea, biuret, and cyanuric acid.

(a)

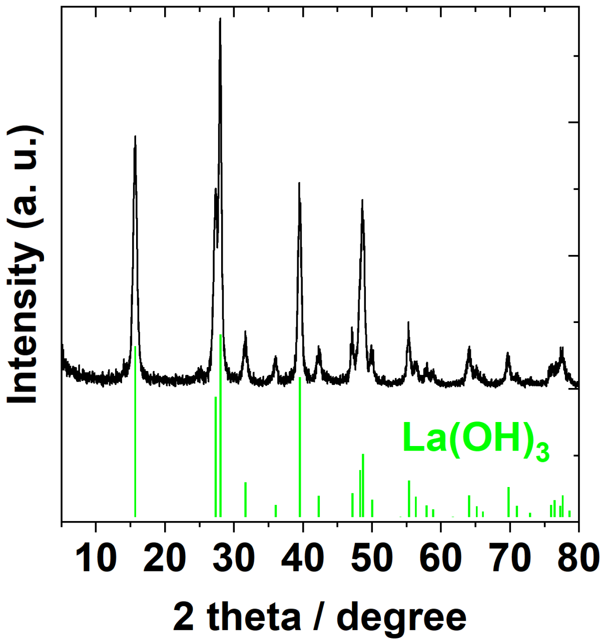

(b)

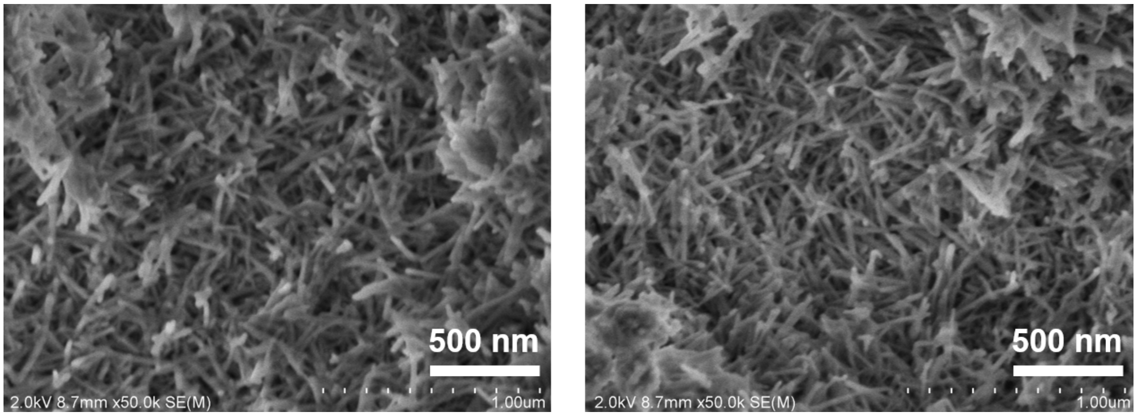

Figure S5. (a) XRD pattern (λ = Cu Kα) and (b) SEM images of $La(OH)_3$ prepared by hydrothermal method.
